# Dendritic and Axonal Wiring Optimization of Cortical GABAergic Interneurons

**DOI:** 10.1007/s12021-016-9309-6

**Published:** 2016-06-27

**Authors:** Laura Anton-Sanchez, Concha Bielza, Ruth Benavides-Piccione, Javier DeFelipe, Pedro Larrañaga

**Affiliations:** 1 Departamento de Inteligencia Artificial, Escuela Técnica Superior de Ingenieros Informáticos, Universidad Politécnica de Madrid, Madrid, Spain; 2 Laboratorio Cajal de Circuitos Corticales, Centro de Tecnología Biomédica, Universidad Politécnica de Madrid, Madrid, Spain; 3 Instituto Cajal, Consejo Superior de Investigaciones Científicas, Madrid, Spain

**Keywords:** Neocortex, Wiring economy, Interneurons, Dendritic and axonal arborizations, Graph theory, Evolutionary computation

## Abstract

The way in which a neuronal tree expands plays an important role in its functional and computational characteristics. We aimed to study the existence of an optimal neuronal design for different types of cortical GABAergic neurons. To do this, we hypothesized that both the axonal and dendritic trees of individual neurons optimize brain connectivity in terms of wiring length. We took the branching points of real three-dimensional neuronal reconstructions of the axonal and dendritic trees of different types of cortical interneurons and searched for the minimal wiring arborization structure that respects the branching points. We compared the minimal wiring arborization with real axonal and dendritic trees. We tested this optimization problem using a new approach based on graph theory and evolutionary computation techniques. We concluded that neuronal wiring is near-optimal in most of the tested neurons, although the wiring length of dendritic trees is generally nearer to the optimum. Therefore, wiring economy is related to the way in which neuronal arborizations grow irrespective of the marked differences in the morphology of the examined interneurons.

**Electronic Supplementary Material:**

The online version of this article (doi:10.1007/s12021-016-9309-6) contains supplementary material, which is available to authorized users.

## Introduction

Santiago Ramón y Cajal formulated the fundamental anatomical principles of the organization of nerve cells more than a century ago. He stated that the structure of axons and dendrites is designed in such a way as to save space, time and matter (Cajal 1899). Here we aim to show that dendritic and axonal trees of different types of cortical interneurons optimize brain connectivity in terms of neuronal wiring cost. Although the concept of wiring cost is not clearly defined, it is basically based on the assumption that the further away two elements are, the more expensive the connection between them is. Therefore, wiring cost can be expressed as a function of the distance between elements, this being the criterion to be minimized.

Wiring cost has been widely used in the literature to explain neuron placement in different brain areas and species, as well as morphological properties in single neurons. Regarding placement, some authors consider the minimization of wiring costs in order to explain neuron placement in simple nervous systems such as *Caenorhabditis elegans* (Kaiser and Hilgetag 2006; Chen et al. 2006; Pérez-Escudero and de Polavieja 2007; Pérez-Escudero et al. 2009). There are also studies on the relation of wiring economy and neuron placement in larger brains. For example, Rivera-Alba et al. (2011) try to explain the placement of neurons in a module of the *Drosophila melanogaster* brain; Chklovskii et al. (2002) associate wiring optimization with the optimal arrangement of elements of neuronal circuits in the mouse neocortex; Kaiser and Hilgetag (2006) further examine the concept of wiring economy analyzing three-dimensional spatial positions of connected cortical areas in the macaque brain; Rivera-Alba et al. (2014) use the concept of wiring economy and the dimensions of neuronal components to predict the microarchitecture of the neuropile across brain areas and species. Karbowski (2015) combines different forms of wiring minimization with the maximization of dendritic spine proportion in the cerebral cortex across species.

Regarding the morphological properties of single neurons, Cuntz et al. (2007, 2008, 2010) and Schneider et al. (2014) use simulations of synthetic neuronal structures to show that optimal wiring explains dendritic branching patterns. Wen and Chklovskii (2008) and Wen et al. (2009) attempt to disclose the relationship between the dimensions and branching structure of dendritic arbors and synaptic distribution by minimizing wiring cost. Other studies formulate mathematically the relation between optimal wiring and different dendritic characteristics. For example, Cuntz et al. (2012) have shown that optimal wiring predicts a 2/3 power law between dendritic wiring length and the number of branching points and also a 2/3 power law between wiring and the number of synapses.

Here we also analyze wiring economy in single neurons. However, we take a different approach from previous research considering a specific criterion of wiring cost assessment, namely, wiring length. We start from the branching and terminal point cloud of real neuronal trees, which we search for the shortest arborization. We force the computed wiring to pass through the branching points to reach the terminal points, and we limit the number of times that the points branch out, since multifurcations rarely occur in real neurons. We hypothesize that by imposing constraints that provide realistic synthetic arborizations, we can for the most part explain the wiring economy of single neurons considering only wiring length. In addition, we search for the longest arborization that meets the same constraints in order to analyze the range of variation of the wiring function. We use the same criteria to analyze both the dendritic and axonal wiring of neurons with very different morphologies.

We use graph theory and evolutionary computation techniques to test our wiring optimization hypothesis. Graph theory is suitable for representing the point clouds and their connections and has been successfully applied in previous works studying dendritic structures (Cuntz et al. 2007, 2008) and neocortical axons (Budd et al. 2010). With the imposed constraints, our wiring design problem is NP-hard, so we had to use heuristic methods for problem solving. We opted for evolutionary computation techniques. Relatively few heuristics have been used to analyze wiring design. For example, Cuntz et al. (2007, 2008) used a greedy algorithm which locally minimizes the total amount of wiring in their synthetic neuronal structures, whereas Pérez-Escudero et al. (2009) and Rivera-Alba et al. (2011) used simulated annealing (Kirkpatrick et al. 1983) to find low-cost neuronal element configurations. To the best of our knowledge, graph theory has not previously been used in conjunction with heuristic methods to analyze both the dendritic and axonal wiring of a set of single neurons with different morphologies. Specifically, we analyze six morphological types of neocortical interneurons, including Martinotti, large basket, common type, horse tail, chandelier and common basket cells (DeFelipe et al. 2013). These interneurons are characterized by different dendritic and axonal morphologies and synaptic connections (see e.g., Ascoli et al. (2008)).

## Methods

### Data

**Fig. 1:**
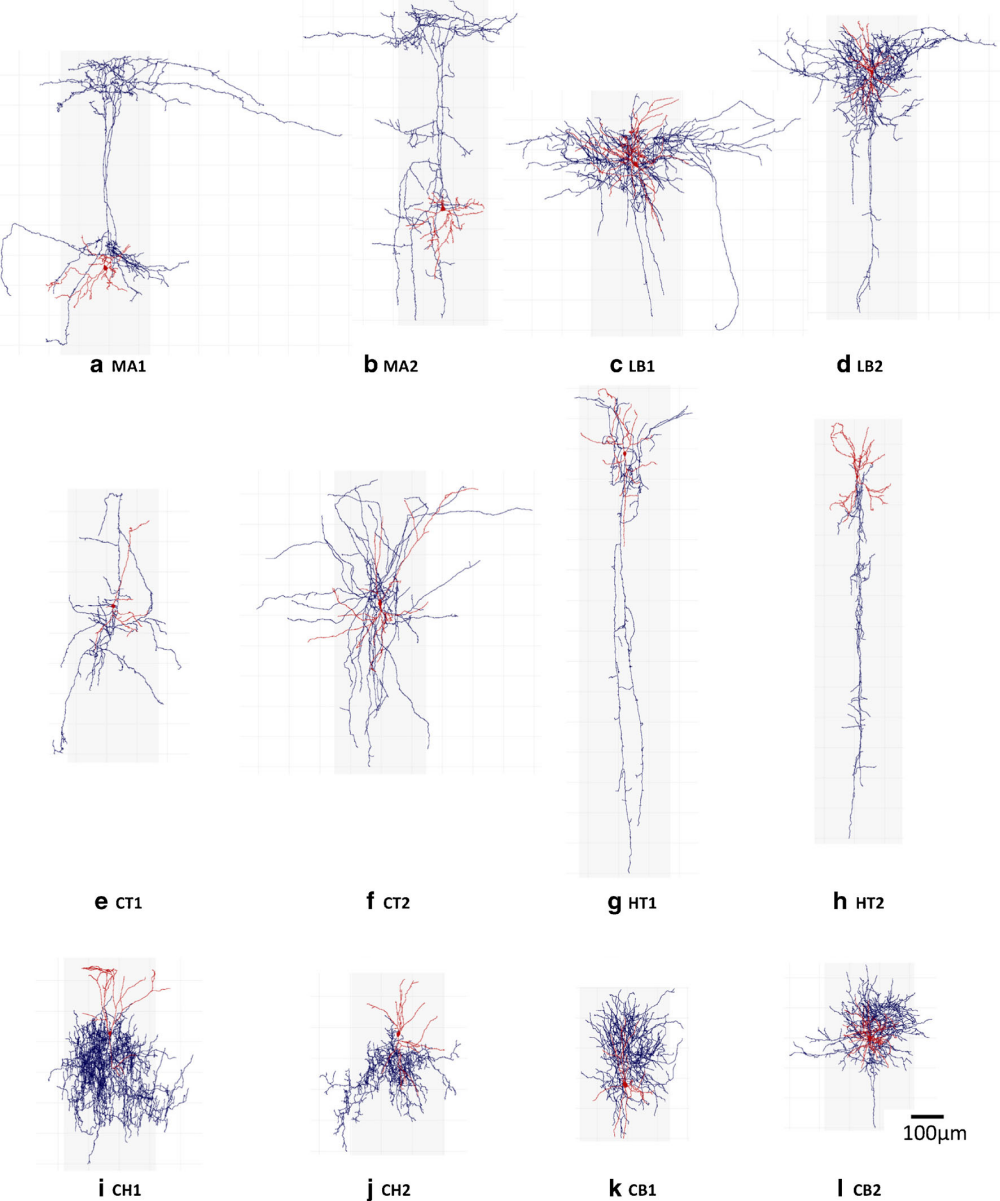
The twelve analyzed interneurons. Dendrites are shown in red and axons in blue. We consider six different types of interneurons depending on their morphology: **a**,**b** Martinotti (*MA*), **c**,**d** large basket (*LB*), **e**,**f** common type (*CT*), **g**,**h** horse tail (*HT*), **i**,**j** chandelier (*CH*) and **k**,**l** common basket (*CB*), as defined in a previous work for the classification on GABAergic interneurons (DeFelipe et al. 2013)

We used a set of 12 three-dimensional reconstructed interneurons (Fig. 1) classified into different types according to their morphology by 42 leading neuroscientists (DeFelipe et al. 2013). These neurons were originally extracted from NeuroMorpho.Org (Ascoli et al. 2007). Table 1 shows the cell type and unique identifier of these neurons in NeuroMorpho.Org. We worked with the following types (two neurons of each type): Martinotti (MA), large basket (LB), common type (CT), horse tail (HT), chandelier (CH) and common basket (CB).

**Table 1:** NeuroMorpho.Org identifier and cell type of the 12 analyzed interneurons. We analyzed the morphology files of the repository version 6.1 (May 2015)

Neuron	NeuroMorpho.Org ID	Type
MA1	NMO_02204	Martinotti
MA2	NMO_00334	Martinotti
LB1	NMO_04572	Large basket
LB2	NMO_04582	Large basket
CT1	NMO_02732	Common type
CT2	NMO_04558	Common type
HT1	NMO_04577	Horse tail
HT2	NMO_00337	Horse tail
CH1	NMO_04548	Chandelier
CH2	NMO_00291	Chandelier
CB1	NMO_01858	Common basket
CB2	NMO_04574	Common basket

### Wiring Algorithm

**Fig. 2:**
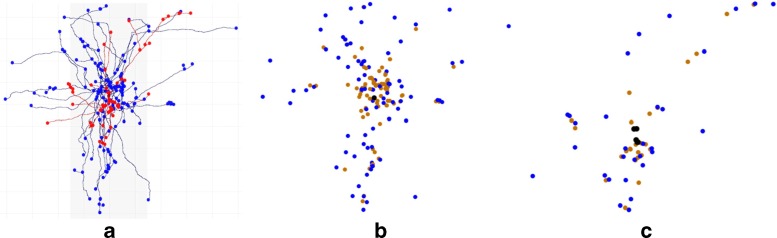
Example of point clouds. **a** Neuron CT2 with superimposed point clouds formed by the roots, branching points and terminal points of the dendrites (*red*) and the axon (*blue*). **b**,**c** Axonal (**b**) and dendritic (**c**) point clouds: the root points are shown in black, the branching points in brown and the terminal points in blue

In general, neurons can be divided into distinct morphological and functional regions: a receptor apparatus (formed by the dendrites and the cell body or soma), the emission apparatus (the axon), and the distribution apparatus (terminal axonal arborization). For example, Fig. 2a shows neuron CT2 in Fig. 1 with superimposed point clouds formed by the roots, branching and terminal points of the dendrites (red) and the axon (blue). We searched for the optimal (the shortest) dendritic wiring from the red point cloud and for the optimal axonal wiring from the blue point cloud. From a graph theory viewpoint, a tree that connects all the target nodes or points together with the minimal cost (length in our case) is called a minimum spanning tree (MST). Well-known classical algorithms exist for building an MST (Kruskal 1956; Prim 1957).

All branching points in the analyzed neurons were bifurcations. Therefore, we forced these nodes to divide into two branches too. In graph theory, the degree of a node is defined as the number of edges incident to it (in our case, the input branch plus the times a point branches out). A tree that connects all points with minimal cost and also limits the degree of each node is called a degree-constrained minimum spanning tree (DCMST). Whereas the MST of a graph is simple to build, finding the DCMST is highly complex (it is an NP-hard problem (Garey and Johnson 1979)). For this reason, a large number of heuristics have been applied in the literature. For example, Krishnamoorthy et al. (2001) compare simulated annealing and genetic algorithms, whereas tabu search is used in Wamiliana (2004), and Bui et al. (2012) propose an ant-based colony algorithm.

In our neuronal wiring analysis, we are looking for minimal cost trees, with constraints on the number of bifurcations. Additionally, to assure that the extent of the dendritic and axonal arborizations is fixed, the roots (i.e., points of origin of the dendrites and axons from the cell body) and terminal points of real neuronal trees should also be unchanged in the searched structures. Therefore, we face DCMST problems where the roles played by the nodes in the trees are also fixed. We can deal with this by building degree- and role-constrained minimum spanning trees (DRCMST) as proposed in Anton-Sanchez et al. (2015). Due to its complexity, DRCMST problem solving is approximated using a wide range of evolutionary computation techniques. The conclusion is that genetic algorithms (Holland 1975), and, in particular, the steady-state genetic algorithm (ssGA) (Syswerda 1991), performed significantly better for the DRCMST problem. Therefore, we solved our neuronal wiring design problems using this technique.

A genetic algorithm mimics the process of natural selection by evolving a population of individuals through random actions that resemble genetic crossovers and mutations. Applying a selection criterion, the algorithm decides which individuals survive (the fittest) and which are discarded. One of the main issues that need to be addressed when using genetic algorithms is the definition and encoding of individuals. In our case, an individual of the population is a feasible neuronal arborization, and each individual is encoded by a permutation as explained below. The smaller the total wiring length is, the fitter an individual is considered to be.

Axonal arborizations consist of a single tree but dendritic arborizations are, generally, formed by a group of trees. The methodology proposed in Anton-Sanchez et al. (2015) can simultaneously optimize one or more trees. Therefore it is applicable to our wiring design problems for both axons and dendrites. Thus, by restricting the number of branches (degree) and the role played by each point in the trees, we search for a single tree with optimal wiring in axonal point clouds and we search for a group of trees with optimal wiring in dendritic point clouds. Then, we compare the resulting structures and the real arborizations. Figure 2b shows the axonal point cloud of neuron CT2 in three different colors, differentiating the three roles with which we work. Figure 2c shows the colored dendritic point cloud. Note that, in this case, we have five roots because the neuron has five dendritic trees (none of the roots are readily appreciable because it is a three-dimensional point cloud).

To search for the optimal arborization that meets the discussed constraints, we formulate and optimize DRCMST problems. As in Anton-Sanchez et al. (2015), an arborization is represented by a permutation of length *n* − *t*, where *n* is the total number of points and *t* is the number of trees to be built. Each position of the permutation represents a connection between two points. The use of two auxiliary arrays of length *n* − *t* to decode the permutation-based representation guarantees degree and role constraints in the trees.

**Fig. 3:**
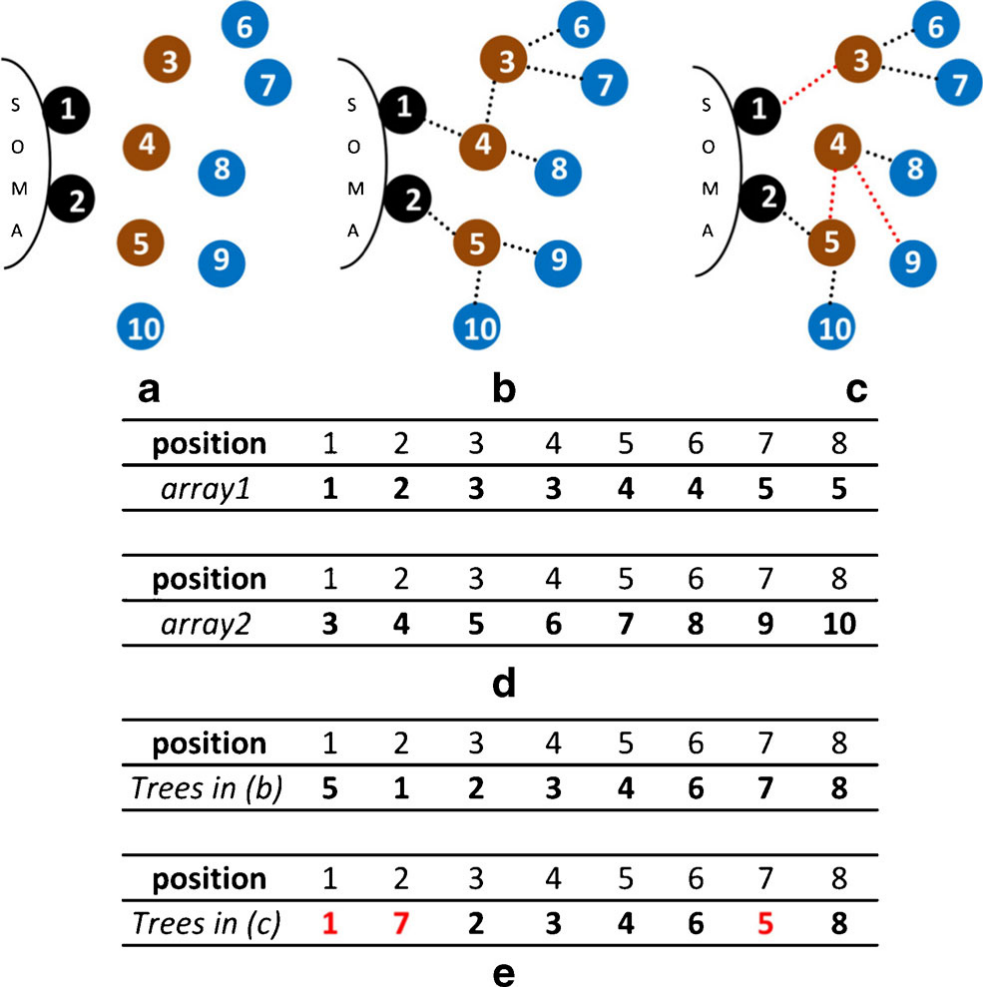
Two examples of dendritic trees of neuron CT2 shown in Fig. 1 and their codification with the proposed permutation-based representation. **a** Numbered point cloud of the two trees. The roots are shown in black, terminal points in blue and branching points (*bifurcations*) in brown. **b** Equivalent structure to real trees. **c** Another valid solution. Differences to (**b**) are shown in red. Note that as the roots are unchanged, the number of constructed trees is always equal to the number of trees in the neuron. However, branching and terminal points from different dendritic trees can be mixed. **d** Auxiliary arrays needed to decode the permutations. **e** Permutations that represent the arborizations in (**b**) and (**c**). Decoding is as follows. A number *s* at position *k* of the permutation means that the node at position *s* of auxiliary array 1 is connected to the node at position *k* of auxiliary array 2. For example, in the permutation shown in (**e**), top, representing arborization (**b**), we find *s* = 5 at position *k* = 1. This means that the node at position 5 in auxiliary array 1 (*node 4*) is connected to the node which is at position 1 of auxiliary array 2 (*node 3*). The number at position *k* = 2 is *s* = 1, which means that the node at position 1 of auxiliary array 1 (*node 1*) is connected to the node at position 2 of auxiliary array 2 (*node 4*), and so on (see Anton-Sanchez et al. (2015) for further details on decoding)

Figure 3 shows an example with two of the dendritic trees of neuron CT2. Figure 3a shows the point cloud of these two trees and uses different colors to identify the roles. Figure 3b shows a solution which matches the real neuronal trees. Figure 3c shows another possible valid set of trees. This small example has *n* = 10 points and *t* = 2 trees. Therefore, the length of the permutations that represent this arborization is *n* − *t* = 8. Figure 3d shows the two auxiliary arrays needed for permutation decoding (Anton-Sanchez et al. (2015) details how these arrays are built). Figure 3e shows the permutations that represent the arborizations in (b) and (c).

The procedure starts with a random initial population of permutations of length *n* − *t*. Then the genetic algorithm performs crossover and mutation operations on the individuals (arborizations) of the population. This results in the generation of new permutations with some changes of position, i.e., with changes to some of the connections that form the trees. The genetic algorithm evolves searching for the arborization with minimal wiring until a stop criterion is met (usually a maximum number of iterations).

### Axon partition

As reported in Anton-Sanchez et al. (2015), DRCMST problems up to 200 nodes can be readily solved. This is the case of dendritic wiring design problems. The computational cost of solving axonal design problems in the same way would be huge because they are much more complex, and it would be very time consuming. Therefore, we introduce parallel computing to address complex problems, that is, we partition the overall axonal point cloud into smaller clouds, and we solve these smaller clouds separately. We can simultaneously solve each of the parts (which takes a few seconds or minutes depending on their size) and then combine the best (shortest) solutions found in each part to ouput the solution that provides the complete axonal tree (negligible time compared to the rest of the process).

**Fig. 4:**
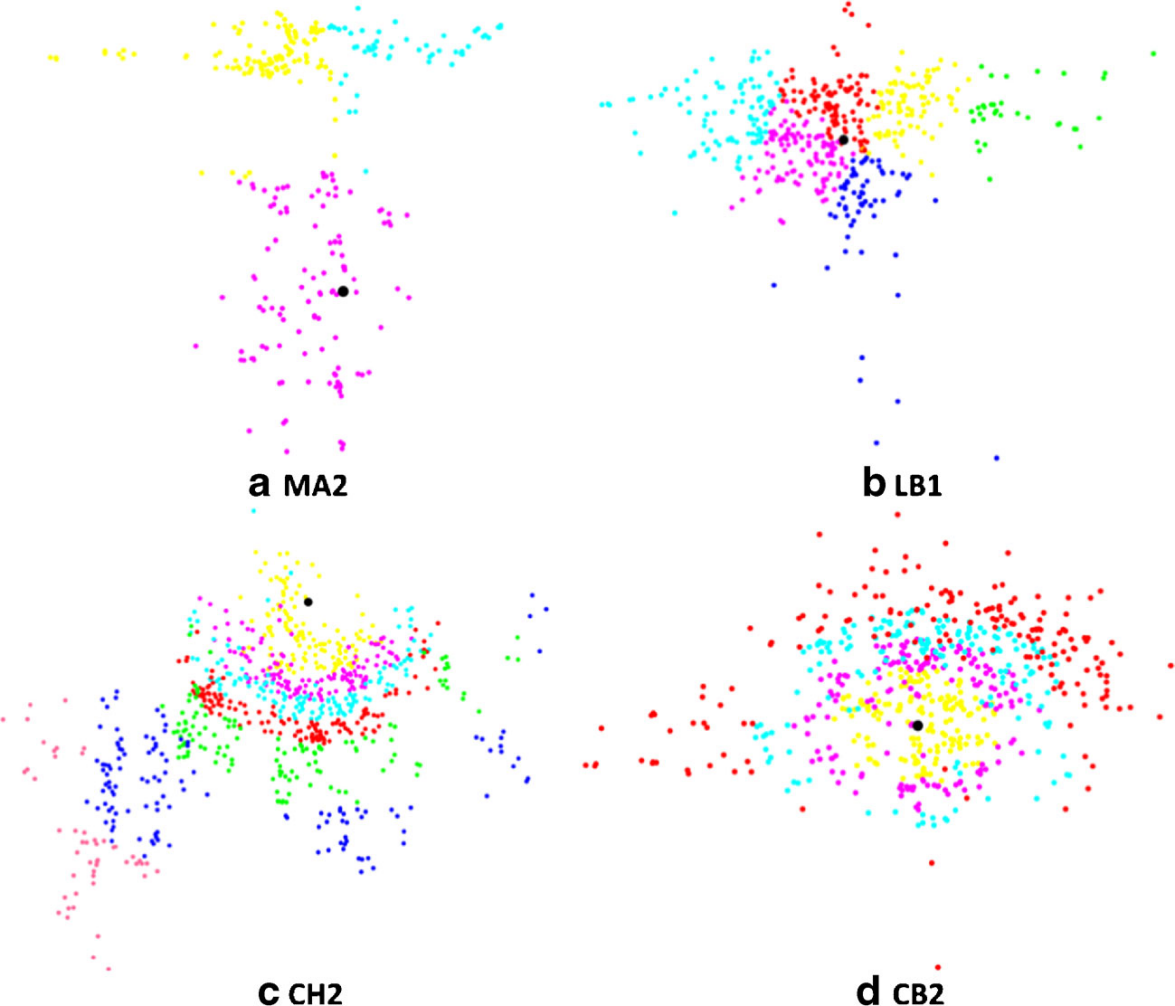
Axonal point clouds of some of the analyzed interneurons divided into smaller clouds to reduce complexity. Sub-regions are shown in different colors and the root of the tree is shown in black. **a** Neuron MA2. 274 points. Groups created using the *k*-means algorithm with *k* = 3 groups. **b** Neuron LB1. 500 points. *k* = 6 groups. **c** Neuron CH2. 800 points. Groups created by distances from nodes to the soma with group size of 125. **d** Neuron CB2. 674 points. Group size of 165

**Fig. 5:**
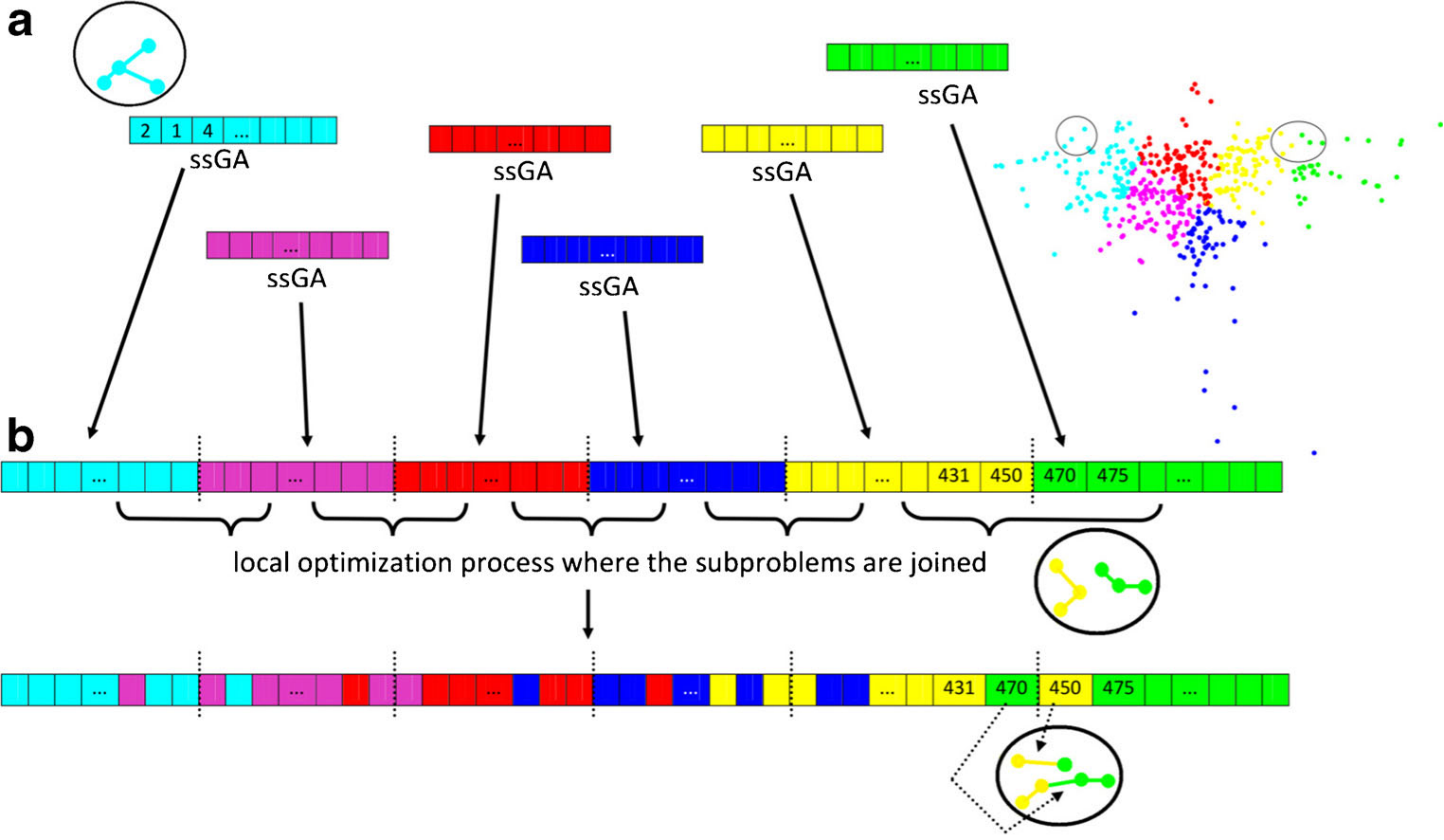
Description of the partitioning process for complex problems with a high number of nodes. Example with the axon of neuron LB1. **a** The axonal point cloud (*500 nodes*) is divided into six smaller point clouds (using the *k*-means algorithm in this case): cyan, magenta, red, blue, yellow and green. The genetic algorithm (ssGA) is applied to each sub-region separately searching for the shortest arborization in each of the smaller point clouds. A sub-region is part of the global permutation depicting the complete axon. Each position of the permutation represents a connection between two nodes (e.g., the first three positions of the cyan permutation correspond to the three connections of the magnified region in this color). **b** We put together the best solutions found in each part to output the global permutation for the complete axonal tree. We apply a local optimization process in the neighborhoods where the sub-region solutions meet: we iteratively switch positions near the junctions of the parts that form the global permutation trying to find better solutions. Switching positions at those permutation locations means changing connections between nearby nodes of two different sub-regions (an example is shown in the magnified region of the yellow and green zones). After the local optimization processes we choose the permutation depicting the best (*shortest*) complete axonal tree. We repeat the procedure in (**a**) and (**b**) 20 times for each neuron (maintaining the same sub-regions). Then we choose and compare with the real axonal tree the best arborization found

The axon is represented by a permutation of length *n* − 1, where *n* is the total number of points in the axonal point cloud. The creation of sub-regions in the overall point cloud is equivalent to partitioning this permutation into as many parts as sub-regions we need to solve. First, we optimize each of the parts into which we divide the permutation, searching for the shortest tree structures in different regions of the point cloud (different colors in Fig. 4). Each sub-region is solved according to the procedure reported in Anton-Sanchez et al. (2015) as described above. Second, we put together the shortest solutions found in each sub-region (sub-part of the global permutation) to output a permutation that represents the entire axonal tree. Third, we try to improve the global solution found. To do this, we iteratively swap permutation positions that are close to the junctions of the parts making up the whole permutation (Fig. 5).

Due to the diversity of axon shapes (spherical, elongated, etc.), we try out two different methods to create the smaller point clouds within the overall set of points. For all the analyzed neurons, we optimize the axonal wiring using the two methods described below. For each neuron, we choose the result provided by the method that performs best, that is, the method that provides the shortest total axonal wiring, and we compare its length with the real axonal wiring.

#### *K*-means algorithm

One method for creating subsets of nodes is the *k*-means unsupervised clustering algorithm (MacQueen 1967) to group nodes according to the distance between them (Figs. 4 a and 4b). We choose a value of *k* nearest to how many hundreds of nodes there are in the point cloud. For example, we choose *k* = 3 for neuron MA2 whose axonal point cloud has 274 nodes.

#### Soma distance

The other method is to form groups of nodes based on their distance to the soma. By setting a group size, e.g. 100, we form the first group with the first 100 nodes of the point cloud that are closest to the soma, the second group with the next 100 nodes closest to the soma, after excluding the nodes used in previous groups, and so on. We test several group sizes for each of the neurons in order to achieve good results for comparison with the real neuronal trees (Figs. 4c and 4d).

### Software

We provide software enabling the user to analyze the wiring optimality of a three-dimensional neuron from its specification in .asc format. The software and a user manual are available for download at the Computational Intelligence Group’s webpage 1 (Software section). It is capable of processing wiring design problems with point clouds up to size 200. Larger problems are costly for a personal computer and are better addressed using parallel computing. Both dendritic and axonal wiring can be analyzed. We implemented the necessary preprocessing for the .asc files in Java and we used the single-objective ssGA implementation provided in jMetal framework (Durillo and Nebro 2011).

## Results

Table 2 summarizes the characteristic features of the 12 neurons analyzed in this study: number of dendritic trees, total number of points (roots, branching and terminal points) of the dendritic point cloud and total number of points of the axonal point cloud (always a single tree). Furthermore, it shows the ratio between the total length of the shortest trees found and the total length of real neuronal trees (see below). The wiring length between two connected points is measured, in both the real and found tree structures, using the Euclidean distance between them. Therefore, we use an approximate real wiring length because we ignore the path tortuosity.

**Table 2:** Characteristics of the 12 interneurons shown in Fig. 1.

	**Dendrites**			**Axon**	
**Neuron**	**Trees**	**Points**	**Best/Real**	**Points**	**Best/Real**
MA1	4	54	**95.50 %**	476	102.59 %
MA2	4	66	**97.33 %**	274	**92.98 %**
LB1	6	100	**93.34 %**	500	**97.98 %**
LB2	7	58	**98.00 %**	822	111.04 %
CT1	3	32	**97.51 %**	236	**96.00 %**
CT2	5	60	**99.91 %**	168	**88.21 %**
HT1	3	44	**98.92 %**	228	**98.12 %**
HT2	2	90	**97.97 %**	156	**86.46 %**
CH1	3	46	**95.24 %**	780	101.36 %
CH2	3	48	**98.59 %**	800	113.01 %
CB1	7	46	**95.29 %**	560	**94.20 %**
CB2	11	132	**91.98 %**	674	109.36 %

### Dendritic Wiring Optimization

The number of dendritic trees in the analyzed neurons varies from 2 to 11 (Table 2); the total number of nodes in these cases is between 32 and 132. For dendritic wiring optimization, we did not apply the partitioning methods described in the previous section because they were not complex problems. The results for dentritic trees are similar across all types of neurons. In all cases, the ssGA algorithm slightly improves upon the real neuronal arborization, i.e., it finds a slightly lower total wiring. The ratio between the length of the best dendritic structure found and the length of the real dendritic trees (fourth column of Table 2) shows that the greatest improvement is achieved for neuron CB2, where the genetic algorithm finds a solution whose total length is 8 % shorter than the real neuronal wiring.

**Fig. 6:**
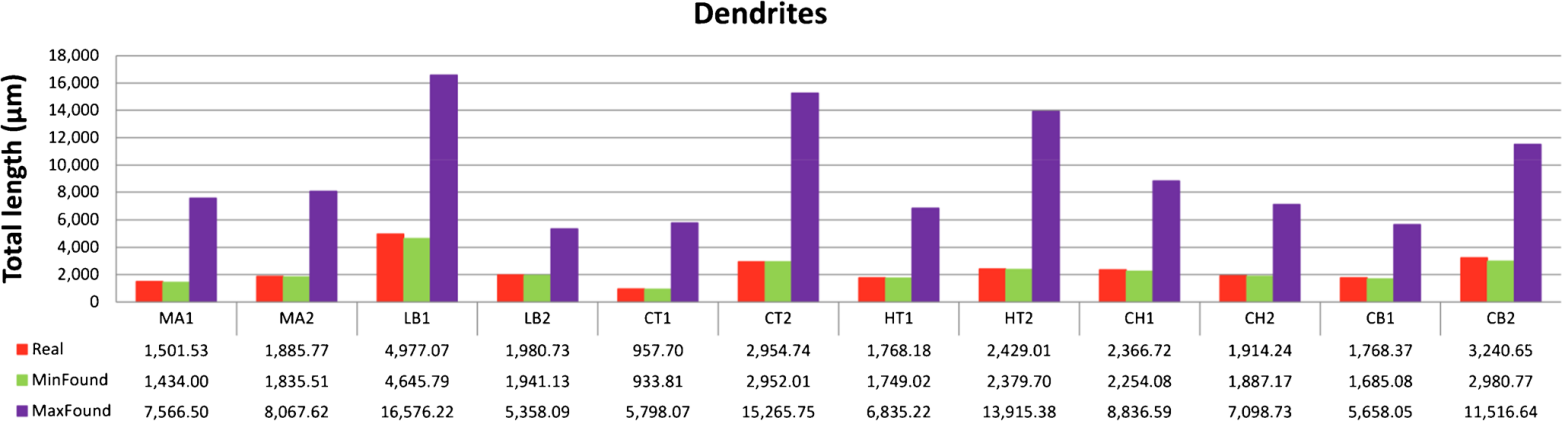
Total dendritic length (*μ*m) of the 12 analyzed interneurons (*red*) versus total length of the minimum and maximum arborizations found (*green and purple, respectively*). In all cases, the optimization algorithm finds a better (*shorter*) solution than the real wiring. The maximum wiring found is much longer than the real wiring (*four times on average*)

To check the range of variation of the wiring function, we performed the optimization process by reversing the direction, that is, we searched for structures that maximized the wiring length while meeting the constraints. As shown in Fig. 6, the maximum wiring of the dendritic arborizations was much longer than the real dendritic wiring (the results ranged from 270.51 % in neuron LB2 to 605.42 % in neuron CT1).

**Fig. 7:**
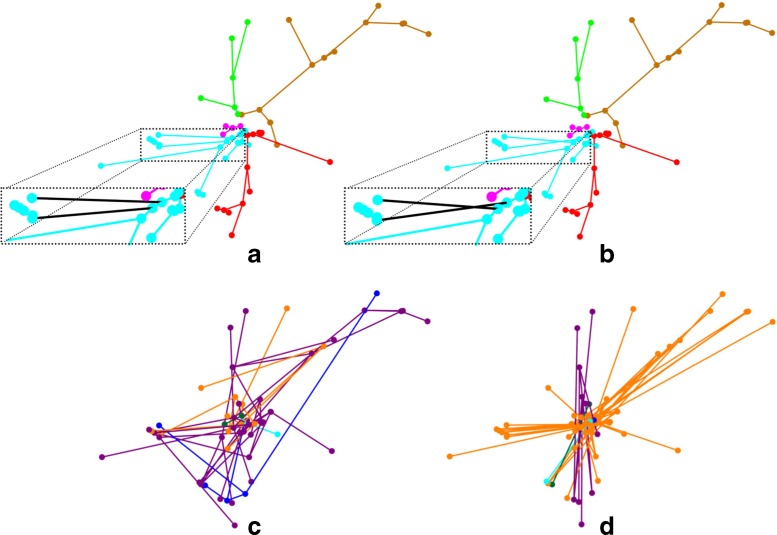
Example of neuron CT2 and differences between real and optimized dendritic wiring. **a** Dendritic point cloud with real connections between points. Five dendritic trees are shown in different colors: brown, green, magenta, cyan and red. **b** Dendritic point cloud with the connections in the shortest structure found by the algorithm. The optimization algorithm finds a structure that improves the real neuronal wiring by only two microns. With the exception of only two edges, the tree structure provided by the algorithm is identical to the real dendritic wiring (black connections in the magnified regions in (**a**) and (**b**)). **c** Structure whose wiring is three times longer than the real wiring. **d** Dendritic point cloud with the connections in the largest structure found, which is five times longer than the real wiring. The trees in (**c**) and (**d**) are very different from the real dendritic trees, and their colors were chosen arbitrarily

Going back to our running example with the dendrites of neuron CT2, Fig. 7 illustrates the difference between the real dendritic wiring and some structures found during the optimization process for the entire dendritic arborization of this neuron. Figure 7a shows the dendritic point cloud with the connections between nodes that exist in the real trees of this neuron. The five dendritic trees of this neuron are shown in five different colors. Figure 7b shows the dendritic connections in the shortest structure found. It is a slight improvement upon the real dendritic wiring. Figure 7d shows the connections of the structure that maximizes the wiring of this neuron. It is more than five times longer than the real wiring (Fig. 6). Figure 7c shows a wiring which is in-between the minimum and maximum found by the optimization algorithm. It is about three times longer than the real wiring. Note that all connections are drawn as straight lines as we measure the (straight) length between points.

### Axonal Wiring Optimization

The axonal point clouds of the 12 analyzed neurons have from 156 to 822 nodes (Table 2) with an average number of nodes greater than 470. As mentioned above, we use two different techniques to create sub-regions in the overall point cloud of each axon to reduce complexity. For each method, we combine the shortest solutions found in each sub-region so that our approach outputs the global minimum arborization (Fig. 5). We choose the result of the technique that returns the shortest total wiring for each neuron.

For Martinotti, large basket and common type neurons (Fig. 1a–f), the best solutions found were clearly better with the *k*-means algorithm (in the case of neuron LB1, there was a 15 % difference in the best solutions found by both methods). For chandelier and common basket neurons (Fig. 1i–l), the best solutions found were vastly better creating groups of nodes depending on their distances to the soma (up to 33 % better than *k*-means algorithm in the case of neuron CB2). For horse tail neurons (Fig. 1g–h), we also achieved better results by grouping the nodes by their distance to the soma. However, the best solutions found for this type of neurons were very similar using both methods.

The results of the two methods used to split the axonal point clouds clearly differentiated which method it is better to apply for each type of neuron. This was predictable considering the shape of the axons. In spherical-shaped axons, like chandelier and common basket neurons, it is better to group the nodes around the root tree. In axons with much less homogeneous shapes, like Martinotti and large basket neurons, it is better to group the nodes taking into account the distance between them regardless of a reference point.

**Fig. 8:**
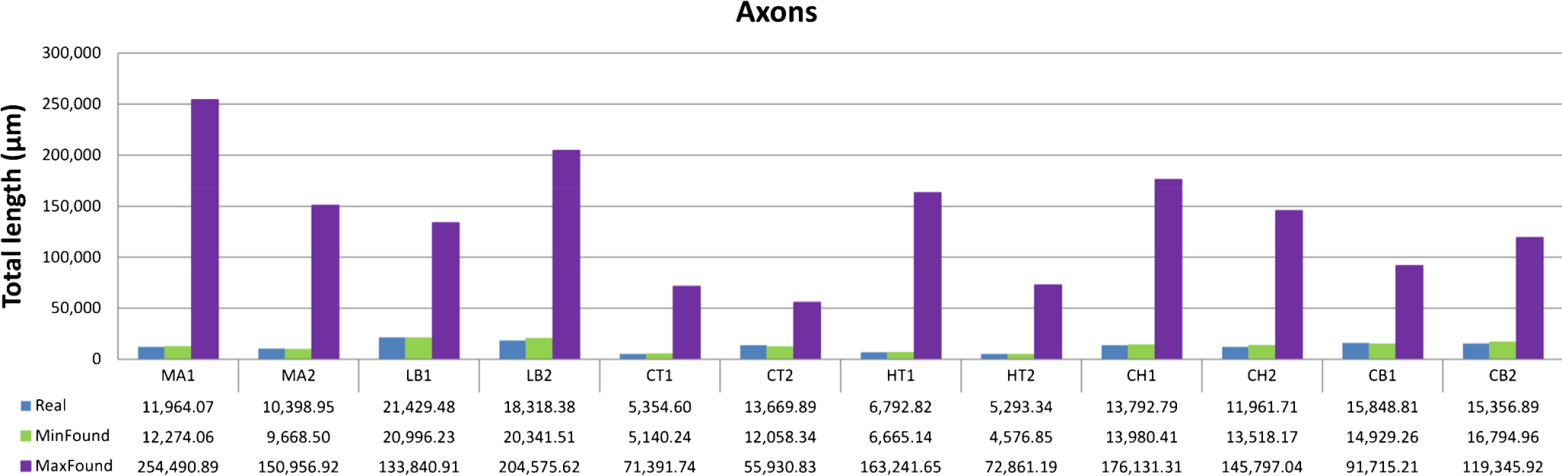
Total axonal length (*μ*m) of the 12 analyzed interneurons (*blue*) versus total length of the minimum and maximum trees found (*green and purple, respectively*). For most axons, the optimization algorithm finds a solution that is shorter than or very close to real wiring. The maximum axonal wiring found is much longer than the real wiring (*12 times on average*)

Unlike dendrites, the tree structures output by the optimization algorithm do not improve upon the real axonal wiring in all cases. In the last column of Table 2, a figure below 100 % shows that the best solution found by the ssGA has a total wiring length shorter (better) than the real axonal tree. A number greater than 100 % indicates that the algorithm cannot find a solution that improves the real wiring. For neuron HT2, for example, we obtain a tree whose total length is almost 14 % less than the real axonal tree. However, for neuron CH2 (one of the most complex axons analyzed with 800 nodes), the best solution found was 13 % worse (longer total length) than the real axonal wiring. We also searched for the trees that maximized the axonal wiring of each neuron. The results varied from 409.15 % in neuron CT2 to 2403.15 % in neuron HT1, i.e., the maximum wiring found was between four and 24 times longer than the real wiring. Figure 8 shows the total real lengths of the 12 axons and the total length of the minimum and maximum solutions found for each neuron.

In some of the axonal wiring design problems, the algorithm was unable to find the real configuration, which was known to exist. Therefore, we performed the following test to check algorithm performance. We generated random point clouds with *n* points and built their MSTs using Prim’s algorithm (Prim 1957). From these MSTs, we constrained the degree and role of each point to match the degree and role in the MSTs. Then, from the original point clouds and with the imposed constraints, we searched for the DRCMSTs. We did this for *n* = 50,100 without problem partitioning. For *n* = 200,400,800, we divided the point clouds into smaller sub-regions using both of the partitioning methods described in Section “Axon partition”.

For small problems, the optimization algorithm was very close to the MST length (2 % larger for *n* = 50 and 6 % for *n* = 100). For larger problems, we applied the partitioning methods to create sub-regions. By optimizing the sub-regions of the point cloud separately, we may not come as near to the global optimum. This is the price we pay for making these problems computationally tractable. For random problems with *n* = 200, the optimization algorithm yielded solutions 14 % larger than the MST length. For *n* = 400,800, the solutions were 23 % and 26 % larger than their MSTs, respectively. For *n* = 200,400, we found the best results, i.e., shortest wirings, creating the sub-regions according to the soma distance. For *n* = 800, the ssGA found the best solutions using the k-means algorithm.

The mean number of points in the 12 dendritic wiring design problems was 65, and the mean best-to-real ratio for the shortest solutions found was 96.63 %. Therefore, we concluded that, because the algorithm performed quite well for similar values of *n*, dendritic wiring was very nearly optimal in terms of wiring length. Comparing axons and dendrites, axonal wiring was not as optimal in terms of wiring length for neurons whose axonal point clouds had the lowest number *n* of points (although *n* was greater than the largest dendritic point clouds). Specifically, the best-to-real ratios in neuron HT2 (*n* = 156) and neuron CT2 (*n* = 168) were 86.46 % and 88.21 %, respectively (Table 2). Neuronal trees appear to expand more optimally in less complex branching structures. Consequently, dendritic wiring, generally simpler than axonal wiring, should come closer to the optimum in terms of the wiring length discussed in this study. In future research, we intend to refine the resolution of large problems in order to explore what happens in the axons for which our algorithm failed to improve upon the real wiring length.

The test that we conducted gives an idea of how well the genetic algorithm performs for problems of different sizes using both partitioning methods, but we must take into account that the comparison of the MST and DRCMST solutions is unfair. The MST for a big point cloud is easily obtainable in polynomial time. However, if the problem has degree and/or role constraints, the problem becomes NP-hard, and large problems are extremely difficult to solve. On this ground, it is necessary to use heuristic methods.

In addition, we extended the study to analyze both the optimality of dendritic and axonal wiring of another 16 neurons (see Supplementary Table 1) to substantiate that the results were similar with groups of neurons with size greater than two (Supplementary Table 2).

## Discussion

We present a new approach to test the hypothesis of optimal neuronal wiring in single neurons using graph theory and evolutionary computation. We analyzed both the dendritic wiring and the much more complex axonal wiring. We found that the tree structure of different types of neocortical interneurons, which included Martinotti, large basket, common type, horse tail, chandelier and common basket cells, is near-optimal in terms of wiring length, although dendritic wiring was generally nearer to the optimum than axonal wiring. This is a remarkable finding since a characteristic of these neurons is that the postsynaptic targets and spatial characteristics of their dendritic and axonal arborizations are rather different (see below). Our analysis stresses the importance of the wiring cost to which some morphological and organizational principles in the brain have been attributed (Chklovskii 2004).

Dendritic wiring optimization was solved properly using the method proposed in Anton-Sanchez et al. (2015). To address axonal wiring design problems, however, we had to reduce their size. The method proposed here is to divide the axonal point cloud into different sub-regions and find the shortest tree structures in each of these sub- regions. The results show that this method performs well in many cases, providing a more efficient method in terms of time and computational cost savings. However, for some of the more complex axons, the optimization algorithm output a tree structure whose total length was close to but larger than real wiring (i.e., the algorithm could not find an equal or better solution than the real situation). Future research needs to improve the way in which the sub-regions are created and how the best solutions found in these sub-regions are combined to output the overall solution. Thus, it would be possible to deal with larger problems.

For all dendrites and many axons, the genetic algorithm (ssGA) used output tree structures with a total length slightly shorter than the real trees. This indicates that dendrite and axon spanning uses the least amount of wiring needed to achieve their functions but that there are also other important factors that influence neuron growth. For example, we might consider a more complete wiring cost function minimizing the distance of each non-root point to the root of the tree. This is closely related to minimizing the time that it takes for a signal to reach a synaptic contact from the soma (see e.g., Cuntz et al. (2007), Wen and Chklovskii (2008), Budd et al. (2010)).

For dendritic trees of the same neuron, we could check if the optimal arborization found has the same number of trees as the real neuron by not fixing the number of trees in advance.

Note also that there are “obstacles”, like blood vessels and cell somata, that the dendrite and axon trajectory has to circumvent. The more such obstacles there are, the greater the wiring cost would be. Moreover, the larger the arbor is, the more the trajectory modifications are. Thus, the wiring may not be perfectly optimal, particularly in axons. However, we did not take tortuosity into consideration (although it would have been more realistic) on the grounds of the complexity of the problem. Moreover, tortuosity is, at least in part, due to the presence of obstacles, and we did not have access to this information. In addition, different types of interneurons connect with different postsynaptic targets, and this is related to the spatial characteristics of their axons. For example, the pattern of postsynaptic contacts may be ‘distributed’ or evenly spaced, whereas others may show a ‘gradient’ pattern where the distribution of contacts changes in a specific direction. ‘Clustered’ terminal branches are characteristic of chandelier cells that innervate pyramidal-cell axon initial segments (see, e.g., Ascoli et al. (2008), Blazquez-Llorca et al. (2014)). Further studies using more complete data on the synaptic characteristics of the cells under study and the local spatial distribution and density of the blood vessels and somata where the neuron is localized will make the wiring rules of single neurons easier to interpret.

## Information Sharing Statement

The source code utilized in this work and a user manual are freely available at http://cig.fi.upm.es/ (Software section).

All the analyzed neurons can be extracted from NeuroMorpho.Org (RRID:SCR 002145, http://neuromorpho.org/) using the identifiers in Table 1 and Supplementary Table 1.

## Electronic supplementary material

Below is the link to the electronic supplementary material.

(PDF 141 KB)

## Footnotes

1 http://cig.fi.upm.es/
