# Supplementary material for: Dendritic and Axonal Wiring Optimization of Cortical GABAergic Interneurons
Source: Neuroinformatics. 2016 Jun 27;14(4):453–64. doi: 10.1007/s12021-016-9309-6 (PMC5010609; doi:10.1007/s12021-016-9309-6)
Supplement: Supplementary file 1 — (PDF 141 KB) [file 12021_2016_9309_MOESM1_ESM.pdf]

Supplementary Material

Neuroinformatics

Dendritic and axonal wiring optimization of cortical GABAergic interneurons

Laura Anton-Sanchez * † Concha Bielza† Ruth Benavides-Piccione‡ §
Javier DeFelipe‡§ Pedro Larrañaga†

1 Analysis of other examples

A broader study analyzed other neurons of two types. In addition to the cells illustrated in Fig. 1, we optimized the wiring of the eight Martinotti cells and the eight large basket cells shown in Supplementary Table 1. These cell types were selected because they are common place in the literature and they have recognizable morphological characteristics (DeFelipe et al, 2013). We applied the k-means algorithm to create the sub-regions in the axonal point clouds of the new neurons because this technique worked best for Martinotti and large basket cells. As shown in Supplementary Table 2, the results for all ten neurons were very similar to what we found for individual neurons (Table 2).

*Corresponding author. Email: l.anton-sanchez@upm.es
†Departamento de Inteligencia Artificial, Escuela Técnica Superior de Ingenieros Informáticos, Universidad Politécnica de Madrid, Madrid, Spain
‡Laboratorio Cajal de Circuitos Corticales, Centro de Tecnología Biomédica, Universidad Politécnica de Madrid, Madrid, Spain
§Instituto Cajal, Consejo Superior de Investigaciones Científicas, Madrid, Spain

Supplementary Table 1. NeuroMorpho.Org identifier of the eight analyzed Martinotti and large basket neurons. Repository version 6.1 (May 2015)

**Martinotti**	**Large basket**
NMO_01848	NMO_00366
NMO_02629	NMO_00293
NMO_01839	NMO_01851
NMO_02203	NMO_04560
NMO_02579	NMO_04581
NMO_02648	NMO_00272
NMO_00306	NMO_00382
NMO_00427	NMO_04576

Supplementary Table 2. Mean number of points ($\bar{n}$) and mean and standard deviation ($\bar{x}_{\pm s}$) of the ratios between the total length of the shortest dendritic and axonal wiring solutions found for each neuron, and the total length of the real trees for ten Martinotti and ten large basket neurons. The results include eight cells of each type (Supplementary Table 1) on top of the two neurons already analyzed in Fig. 1

	**Dendrites**		**Axon**	
**Type**	$\bar{n}$	$\bar{x}_{\pm s}$	$\bar{n}$	$\bar{x}_{\pm s}$
**Martinotti**	81.0	$96.66\%_{\pm 2.2\%}$	527.2	$101.58\%_{\pm 10.7\%}$
**Large basket**	53.4	$97.81\%_{\pm 1.8\%}$	488.4	$104.85\%_{\pm 14.1\%}$

References

DeFelipe J, Lopez-Cruz P, Benavides-Piccione R, Bielza C, Larrañaga P, Anderson S, Burkhalter A, Cauli B (2013) New insights into the classification and nomenclature of cortical gabaergic interneurons. Nature Reviews Neuroscience 14(3):202–216
